# Camera traps and guard observations as an alternative to researcher observation for studying anthropogenic foraging

**DOI:** 10.1002/ece3.8808

**Published:** 2022-04-13

**Authors:** Ben J. Walton, Leah J. Findlay, Russell A. Hill

**Affiliations:** ^1^ 56868 Department of Anthropology University of Durham Durham UK; ^2^ Primate & Predator Project Lajuma Research Centre Louis Trichardt South Africa; ^3^ 56868 Department of Zoology University of Venda Thohoyandou South Africa

**Keywords:** Chlorocebus, crop foraging, crop‐raiding, human–wildlife conflict, human–wildlife interactions, Papio

## Abstract

Foraging by wildlife on anthropogenic foods can have negative impacts on both humans and wildlife. Addressing this issue requires reliable data on the patterns of anthropogenic foraging by wild animals, but while direct observation by researchers can be highly accurate, this method is also costly and labor‐intensive, making it impractical in the long‐term or over large spatial areas. Camera traps and observations by guards employed to deter animals from fields could be efficient alternative methods of data collection for understanding patterns of foraging by wildlife in crop fields. Here, we investigated how data on crop‐foraging by chacma baboons and vervet monkeys collected by camera traps and crop guards predicted data collected by researchers, on a commercial farm in South Africa. We found that data from camera traps and field guard observations predicted crop loss and the frequency of crop‐foraging events from researcher observations for crop‐foraging by baboons and to a lesser extent for vervets. The effectiveness of cameras at capturing crop‐foraging events was dependent on their position on the field edge. We believe that these alternatives to direct observation by researchers represent an efficient and low‐cost method for long‐term and large‐scale monitoring of foraging by wildlife on crops.

## Graphical abstract

Understanding anthropogenic foraging by researcher observation is costly and labor‐intensive. We investigated whether camera traps and crop guard records could give the same insights as researcher observation into patterns of crop‐foraging by baboons and vervet monkeys. Camera and guard data predicted data from researchers for baboons, and to an extent, vervets, and therefore present a viable alternative to researcher observation allowing for large‐scale and long‐term monitoring of crop‐foraging.

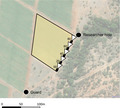

## 1 INTRODUCTION

A wide variety of large vertebrate taxa forage on anthropogenic food sources (Hill, 2018). This can have significant socioeconomic impacts on both urban and rural communities (Haule et al., 2002; Hill & Wallace, 2012; Kagoro‐Rugunda, 2004; Mwakatobe et al., 2014; Nyhus et al., 2005), negatively affecting attitudes toward wildlife and conservation among local people (Findlay, 2016; Nyhus et al., 2000). Animals deemed to be problematic may be translocated (Imam et al., 2002; Strum, 2005) or killed (Findlay, 2016; Hill, 2000; Hoare, 2015; Mwakatobe et al., 2014), with implications for wildlife conservation. Feeding on anthropogenic foods can also significantly alter animal behavior (Beckmann & Berger, 2003; Fehlmann, O'Riain, Kerr‐Smith, Hailes, et al., 2017; Lowry et al., 2013; Walton et al., 2021). These effects on wildlife and humans are often conceptualized as “human‐wildlife conflict” (Hill, 2021).

Direct observation by researchers can provide a detailed understanding of the behavior of animals foraging on anthropogenic foods and this understanding can be used to alleviate negative impacts on both people and wildlife (Fehlmann, O'Riain, Kerr‐Smith, Hailes, et al., 2017; Findlay & Hill, 2021a; Hockings et al., 2009; Wallace, 2010). Researchers can use a range of visual and auditory cues to detect animals engaging in anthropogenic foraging and record details of their behavior and while no method is completely accurate, direct observation is often the default strategy for data collection on the behavior of wild animals. However, behavioral observation is also costly and time‐consuming, which can limit its use, especially over large areas, or long time periods. Furthermore, the presence of researchers is unlikely to be neutral and can have unintended influences on animal behavior (Allan et al., 2020; McDougall, 2012; Nowak et al., 2014). There is thus a need for alternative, lower cost methods for understanding patterns of animal crop‐foraging that can extend across larger temporal and spatial scales and from which robust conclusions can still be drawn.

Camera traps provide a potential alternative. They are relatively cheap, require little expertise to set up and maintain, and can be deployed for long periods of time while recording data day and night, seven days a week, which is difficult for researchers to achieve through observation (Pebsworth & LaFleur, 2014). Some animals crop‐forage by night (Gunn et al., 2014; Krief et al., 2014), behavior which is particularly challenging to study through observation. Camera traps also avoid observer bias in data collection and have a lower impact on species behavior (Caravaggi et al., 2020). Use of machine learning (Tabak et al., 2019) and citizen science (Swanson et al., 2015), or combinations of the two (Green et al., 2020; Willi et al., 2019), is reducing the time required for tagging images and data analysis is becoming simpler with packages like CamtrapR (Niedballa et al., 2016) providing full image to analysis workflows. Camera traps have already been used to answer questions about animals foraging on anthropogenic sources, providing data on the species (Abrahams et al., 2018; Findlay, 2016), numbers, identity, age, and sex distribution of foraging animals (Ranjeewa et al., 2015; Smit et al., 2019), as well as the effectiveness of deterrents (Branco et al., 2019; Findlay & Hill, 2021b; Ngama et al., 2018; Pozo et al., 2019; Ranjeewa et al., 2015), and the diurnal (Findlay & Hill, 2021a; Ranjeewa et al., 2015; Smit et al., 2019; Zak & Riley, 2017) and seasonal timings of crop‐foraging (Zak & Riley, 2017). However, where camera traps have been used to assess crop‐foraging in the past, what they can and cannot measure is often assumed. There has been little effort to date to establish whether camera traps can reliably record patterns of crop‐foraging, as characteristics of species, such as body size or gregariousness, may create systematic biases in camera trap data leading to erroneous conclusions (Kolowski & Forrester, 2017; Pebsworth & LaFleur, 2014; Treves et al., 2010).

Guards or rangers often work to prevent animals accessing anthropogenic foods and could also collect data. In Cape Town, South Africa, rangers deter baboons from entering urban areas (Bracken et al., 2021; Fehlmann, O'Riain, Kerr‐Smith, & King, 2017a). Subsistence farmers may chase any animals that attempt to forage on their crops (Hill, 2018) and in large‐scale agriculture, farmers may hire crop guards to chase animals (Findlay & Hill, 2021b). These people regularly encounter animals foraging on anthropogenic foods. Recordings taken by farmers, guards, or rangers on the species, timings, and number of animals consuming anthropogenic foods can inform the management of wildlife crop‐foraging. Furthermore, participatory methods can encourage engagement in wildlife conservation (Marks, 1994). Previous research has relied on interviews (Abrahams et al., 2018; Giefer & An, 2020; Sekhar, 1998; Spagnoletti et al., 2017; Webber & Hill, 2014; Zak & Riley, 2017), but few studies have used data collected by farmers, guards, or rangers at the very moment crop‐foraging happens (Linkie et al., 2007; Nyhus et al., 2000). Those that have, have generally done so without considering possible biases and limitations of this method. For example, those working on crop fields have other jobs to do so data recording is unlikely to be a priority; when animals enter a field, farmers are likely to chase the animals, rather than record the exact time they entered the field. Guards may also have large fields to protect, making it more likely that they miss crop‐foraging animals.

We previously used approximately 900 hours of direct observational data to explore patterns of crop‐foraging by unhabituated chacma baboons (*Papio ursinus*) and vervet monkeys (*Chlorocebus pygerythrus*) on a commercial farm in the Limpopo province of South Africa (Findlay & Hill, 2021a). Baboons and vervets are often cited as two of the most problematic crop‐foraging animals in the area and are regularly shot and killed by farmers (Findlay, 2016). Baboons caused more crop loss than vervets, foraged on crops more in the mornings, and their rates of crop‐foraging increased when plant primary productivity on the study farm was low. Vervet monkey rates of crop‐foraging were primarily influenced by the presence of baboons, but they did not show any clear temporal patterns.

While these observational data were being recorded, two other sources of data were collected for the same field over approximately the same period. A crop guard recorded the timings of crop‐foraging events by vervet monkeys and baboons and five camera traps recorded images of animals entering and exiting the field. Here, we investigate how well measures from these two methods predict those from researcher observation to understand the strengths and weaknesses of cameras and guards for assessing patterns of crop‐foraging. While data collected by researcher observation cannot give a perfect representation of crop‐foraging, we believe that the methods used in our study (studying a small area and taking measures to reduce researcher fatigue) mean the data can be treated as a reliable baseline.

## 2 MATERIALS AND METHODS

### 2.1 Study location

**FIGURE 1:**
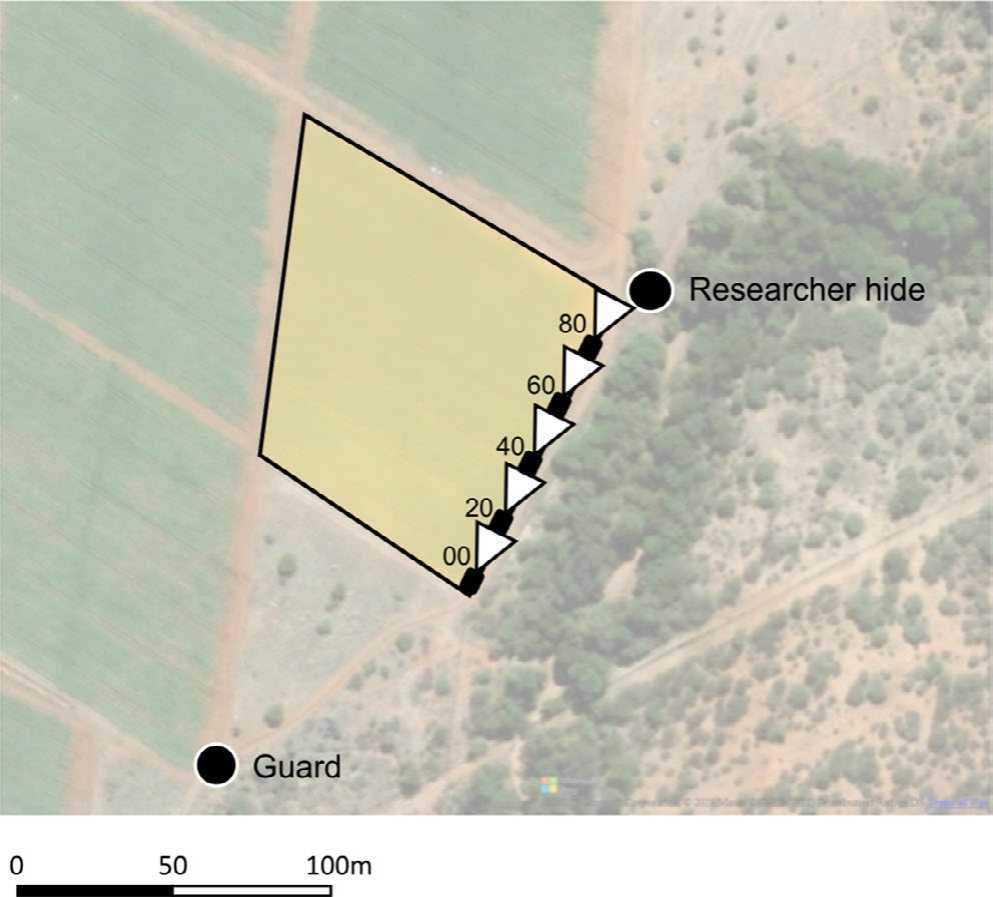
Map of the study field (highlighted in yellow). Camera trap locations are marked in black, with the field of view indicated in white. Numbers beside each camera represent the distance from the southerly corner of the field in meters. The locations of the field guard station and the researcher hide are also marked

We collected data on a commercial farm in the Blouberg District Municipality, in the north of the Limpopo Province, South Africa. The climate is semi‐arid, and the area is prone to frequent drought. The vegetation surrounding the study farm is Limpopo Sweet Bushveld (Mucina & Rutherford, 2006). The region is an important area for crop production in South Africa, producing tomatoes, potatoes, onions, beans, pumpkins, squashes, melons, citrus fruits, and tobacco. The study farm was selected because it had a history of crop‐foraging and was typical for the area in terms of crops grown and mitigation activities. Vertebrate species known to forage on crops in the region include vervet monkeys, chacma baboons, porcupine, bushpig, warthog, and antelope species such as bushbuck and common duiker (Findlay, 2016). Crop‐foraging chacma baboons and vervet monkeys had been shot on the farm for several years prior to the research (personal communication), a common strategy of control in the region (Findlay, 2016). Like many other farms in the area, the study farm employed field guards to protect crops. These guards, normally women, were present at fields seven days a week during daylight hours and shouted, chased, and threw stones at wildlife entering crop fields to forage (Findlay & Hill, 2021b). We collected data on a single 1‐ha field that the farmer felt had been most impacted by crop‐foraging in the past, close to natural vegetation and a river (Figure 1). The farmer planted butternut squash in this field on 29/01/2013 and harvested the crop from the end of June to 20/08/2013. Butternut is a common crop in the region and food preference trials have indicated that it is selected by chacma baboons over most other crop items grown in the region (L. J. Findlay, unpublished data).

### 2.2 Researcher observational data collection

Data collected by researchers have previously been reported in Findlay and Hill (2021a). Researchers were concealed in a hide in one corner of the field, on the side adjacent to the natural vegetation (Figure 1). It was thought that this was the side of the field that primates were most likely to use to enter the crops. Though the presence of the researchers concealed in a hide cannot be considered neutral, they did not elicit any significant alarm calls, vigilance, fleeing, or avoidance behaviors. Furthermore, the hide was present on the field for two weeks prior to data recording to allow primates to habituate to it. The focal field was relatively small (1 ha), and therefore, researchers could easily see and hear any primates entering the field, meaning it was unlikely that researchers would miss primate crop‐foraging events. To prevent researcher fatigue, which could reduce the quality of data collected, observers changed over between the morning (06:00–12:00) and afternoon (12:00–18:00). Foraging events were recorded using a Canon Legria HFR506 video camera and foraging events were live coded. A crop‐foraging event was defined as starting when the first individual of a group entered the field and finishing when the last individual left the field. One minute had to pass with no animals in the field for another entry into the field to be classed as a separate crop‐foraging event. When more than one species was recorded in a field, separate crop‐foraging events were recorded for each individual species. Observations were recorded from 01/05/2013 to 20/08/2013 over approximately five days per week between dawn and dusk (approximately 06:00–18:00).

For each crop‐foraging event, the following were recorded: species, time when the first individual entered the field, time when the last individual exited the field, and the number of butternut squash each individual carried out of the field. This gave the timings of crop‐foraging events and the number of crop items removed in each crop‐foraging event, an estimate of crop loss. This measure will be an underestimate of crop damage, as it does not account for crop loss due to consumption or damage in the field. It was not possible to fully estimate crop loss, as researcher presence in the field to assess damage may have altered primate behavior.

### 2.3 Guard observational data collection

One guard was located next to the focal field (Figure 1) with the job of protecting this field and three neighboring fields from crop‐foraging animals. Fields were close enough together for the guard to monitor and easily access all four fields. They were given a notebook and pen and asked to make notes when animals came into the studied crop field. Specifically, they were asked to record the date, time, and species. A crop‐foraging event was defined in the same way as for researcher observation, starting when the first individual of a group entered the field and finishing when the last individual left the field. One minute had to pass with no animals in the field for another entry into the field to be classed as a separate crop‐foraging event. When more than one species was recorded in a field, separate crop‐foraging events were recorded for each individual species. The information requested was limited to ensure that data recording was not too onerous for field guards. Researchers checked in with the guard at regular intervals to ensure data were recorded. Data were collected from 07/02/2013 to 11/08/2013. The guard worked from sunrise to sunset, approximately 06:00–18:00. As vervets and baboons are diurnal (Ayers et al., 2019; Isbell et al., 2017), this covered the times that these species were likely to crop forage.

### 2.4 Camera trap data collection

Five motion‐triggered camera traps (Bushnell 2010 Trophy Cameras) were set up at 20‐m intervals along the edge of the field adjacent to natural vegetation, facing along the line of the fence (Figure 1). The farmer had observed that this edge was most used by animals entering to crop‐forage and it was hoped these cameras should capture most crop‐foraging events. Camera traps were tested for appropriate spacing by a researcher walking past in a crouched position; 20‐m intervals recorded all movements between cameras. Cameras collected images in a three‐shot burst. After a camera was triggered, there was a five‐second rest period during which a camera could not be triggered. Images were coded for species, date, and time. Cameras collected data from 08/03/13 to 03/10/13 across the 24‐h period.

All data collection was approved by Durham University's Animal Welfare Ethical Review Board (formerly Life Sciences Ethical Review Process Committee) and a permit issued from the Limpopo Department of Economic Development, Environment and Tourism. Approval for research involving the guards was given by the Department of Anthropology Ethics Subcommittee at Durham University.

### 2.5 Analysis

We conducted statistical analyses for the period in which all three datasets overlapped: 01/05/2013 to 08/08/2013. To maximize the available dataset, we simulated how many crop‐foraging events were recorded by camera subsets for the entire timeframe that camera data were available.

We extracted two measures of crop‐foraging from the data. Firstly, we recorded the number of daily “crop‐foraging events,” with a crop‐foraging event defined as when vervets or baboons were detected in the field by any of the three methods. While the presence of primates in the field does not necessarily mean crop‐foraging was taking place, researchers did not observe any times when primates were inside the field without crop‐foraging. Furthermore, using the term “crop‐foraging event” to refer to primates in the field maintains consistency with definitions used previously (Findlay & Hill, 2021a, 2021b). For the camera traps, we classed photographs as a new crop‐foraging event if at least 30 min had passed since the previous photograph of the same species on any of the five cameras, to reduce issues of temporal dependence. Behavioral observations showed that crop‐foraging events by vervets and baboons were up to, but rarely greater than, 30 min and so this was deemed appropriate to delineate separate crop‐foraging events. We classed images of different species within 30 min of one another as separate crop‐foraging events. We used the package CamtrapR to remove temporally dependent images (Niedballa et al., 2016). Researchers recorded the number of crop items removed during each crop‐foraging event, as a proxy for crop loss, the parameter most relevant to farmers.

We divided the study into ten‐day periods, as it is unlikely that data recorded on a smaller timescale than ten days would translate into practical recommendations that can be acted upon by farmers. We calculated the mean daily value of each measure within a ten‐day period for the days where data were recorded for all three methods, as researchers were not present on all days. For example, if researchers were absent for two days in a ten‐day period, then we removed guard and camera data for the same days and calculated a mean for the eight remaining days. As researchers were not able to record the number of crop items removed for some crop‐foraging events, we excluded these data from analysis on crop items. However, the same crop‐foraging event could contribute to the overall number of crop‐foraging events.

We used a linear regression analysis to assess the extent to which data collected by crop‐guards and cameras predicted data collected by researchers, specifically: (a) whether the number of crop‐foraging events recorded by guards and cameras predicted the number of crop‐foraging events recorded by researchers, and (b) whether the number of crop‐foraging events recorded by guards and cameras predicted the number of crop items removed from the field as recorded by researchers.

We also simulated the number of independent crop‐foraging events that would have been recorded by subsets of our five camera locations. For all combinations of one, two, three, and four camera locations, we first removed temporally dependent images (photographs within 30 min of each other) to create new simulated datasets of independent crop‐foraging events. There were five possible combinations of four cameras, ten of three cameras, ten of two cameras, and five of one camera resulting in 30 simulated datasets. Here, we report the number of crop‐foraging events that would have been recorded for each simulated subset of the cameras originally deployed.

## 3 RESULTS

### 3.1 Crop loss and crop‐foraging events

We used a linear regression analysis to investigate the extent to which data from guards and cameras predicted data on crop‐foraging as recorded by researchers (Table 1). For baboons, the number of crop‐foraging events recorded by guards and by cameras predicted crop loss recorded by researchers. The number of crop‐foraging events recorded by guards and cameras also predicted the number of crop‐foraging events recorded by researchers (Table 1).

For vervets, the number of crop‐foraging events recorded by guards predicted crop loss recorded by researchers, but the number of crop‐foraging events recorded by cameras did not. The number of crop‐foraging events recorded by guards and cameras predicted the number of crop‐foraging events recorded by researchers (Table 1).

**TABLE 1:** Summary statistics from linear regression analyses assessing how well guard and camera data predict researcher data

Predictor	Response	Species	Adj. *R* ^2^	Residual SE	*F* statistic	*p*‐Value
Guard recorded crop‐foraging events	Researcher recorded crop loss	Baboons	0.61	10.7	14.9	.005^*^
Camera recorded crop‐foraging events	Researcher recorded crop loss	Baboons	0.80	7.63	36.9	<.001^*^
Guard recorded crop‐foraging events	Researcher recorded crop‐foraging events	Baboons	0.45	2.29	8.49	.020^*^
Camera recorded crop‐foraging events	Researcher recorded crop‐foraging events	Baboons	0.90	0.99	78.6	<.001^*^
Guard recorded crop‐foraging events	Researcher recorded crop loss	Vervets	0.54	1.58	11.4	.010^*^
Camera recorded crop‐foraging events	Researcher recorded crop loss	Vervets	0.25	2.00	4.04	.079
Guard recorded crop‐foraging events	Researcher recorded crop‐foraging events	Vervets	0.69	0.77	21.1	.002^*^
Camera recorded crop‐foraging events	Researcher recorded crop‐foraging events	Vervets	0.41	1.06	7.19	.028^*^

*p < .05.

### 3.2 Optimizing camera trap numbers

**FIGURE 2:**
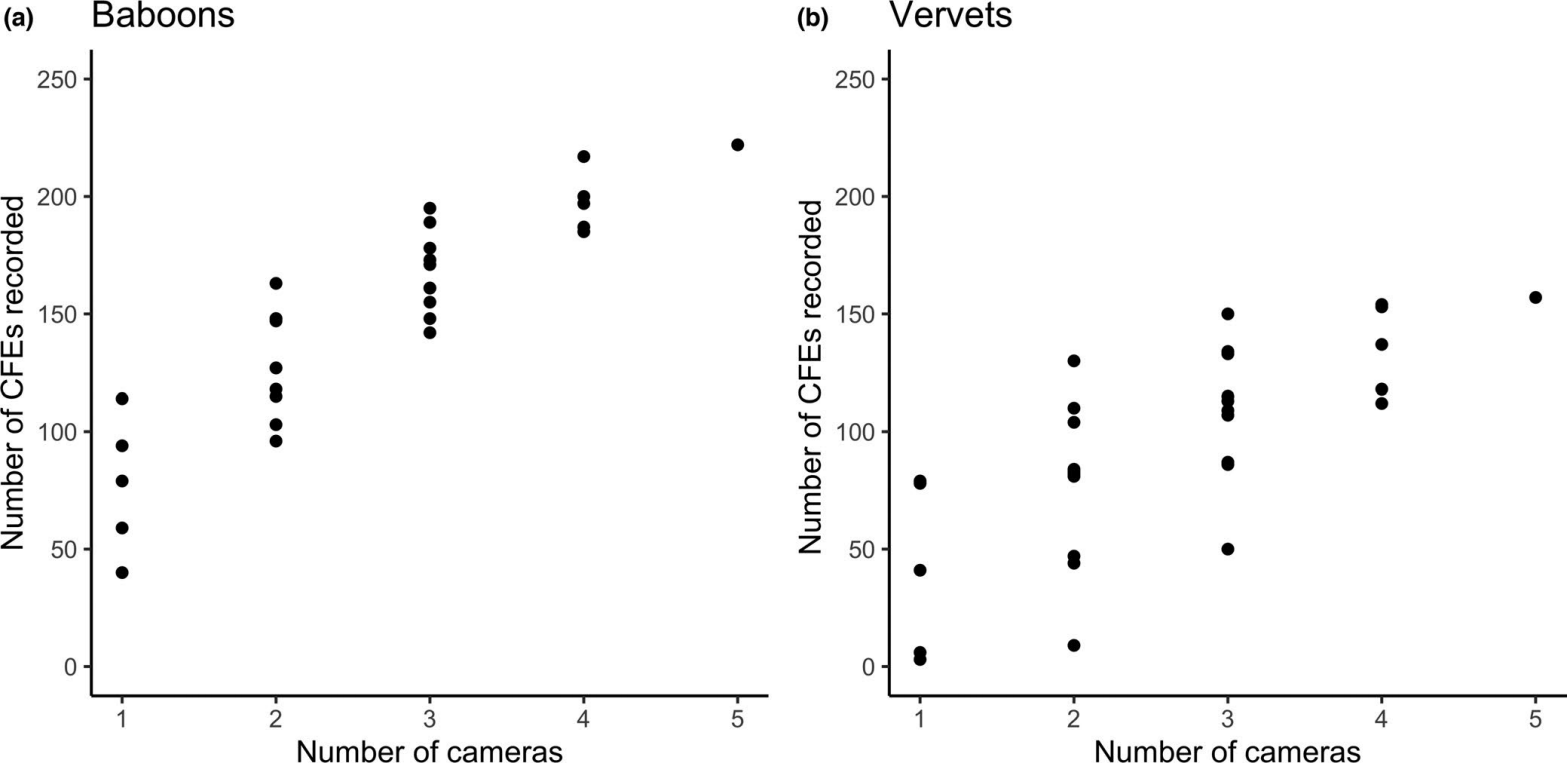
Number of independent crop‐foraging events (CFEs) recorded by different numbers and combinations of camera traps for (a) baboons and (b) vervet monkeys

**FIGURE 3:**
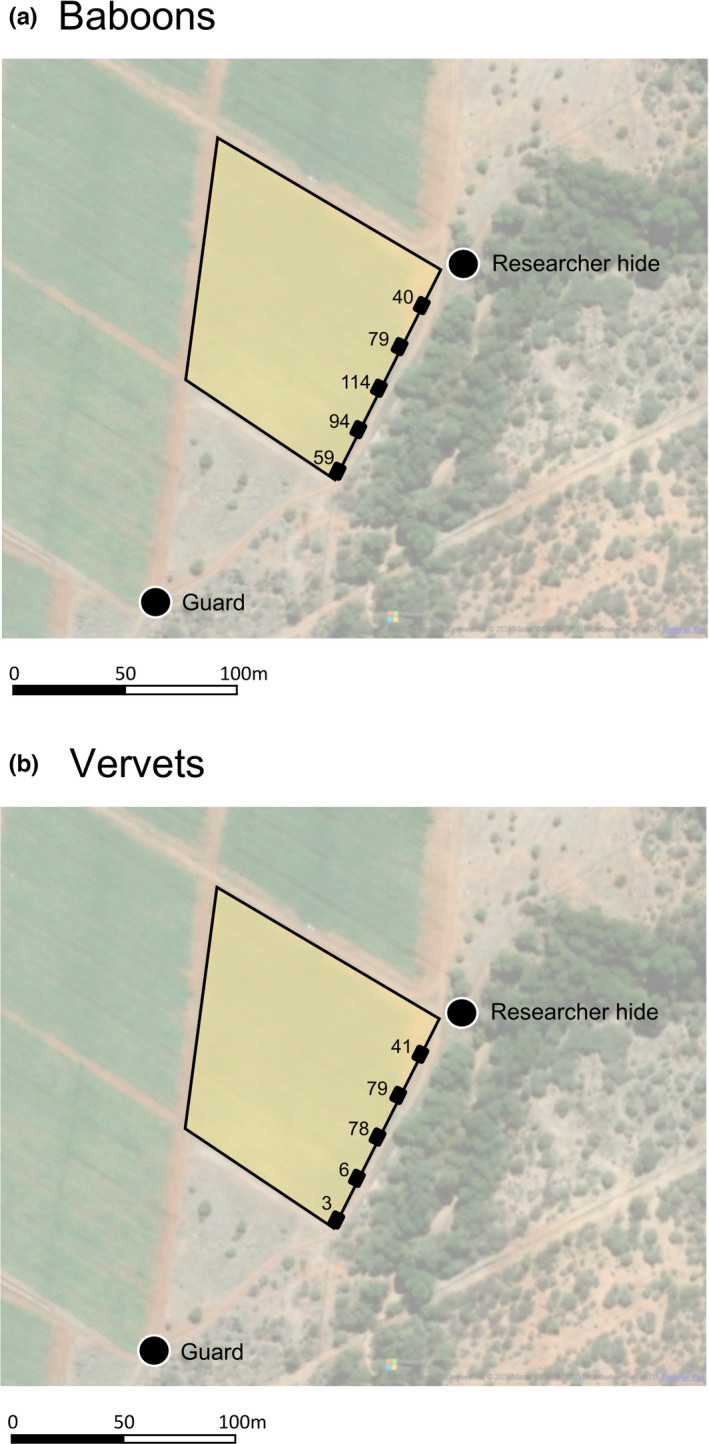
Map showing the number of crop‐foraging events recorded by each camera alone for (a) baboons and (b) vervet monkeys, and the position of the researcher and guard on the field

We determined the number of crop‐foraging events that would have been recorded if there were fewer cameras than the five originally deployed and how their location in the field would have affected the number of crop‐foraging events recorded. We simulated all possible combinations of one, two, three, four, and five cameras by resampling from the five originally deployed. The number of crop‐foraging events recorded generally increased with the number of camera traps (Figure 2), but the number of crop‐foraging events recorded by each camera varied depending on their location in the field (Figure 3).

## 4 DISCUSSION

Our results show that data collected by guards and cameras can predict crop loss and the frequency of crop‐foraging events as recorded by researchers for baboons and to an extent, for vervets. The number of crop‐foraging events recorded by guards and cameras predicted crop loss and the number of crop‐foraging events recorded by researchers for baboons, with cameras the best predictor of both crop loss and the number of baboon crop‐foraging events. This was not the case for vervets, where crop‐foraging events recorded by cameras and guards only weakly predicted the same parameter by researcher measures and only crop‐foraging events recorded by guards predicted crop loss as recorded by researchers; data from cameras did not. The number of crop‐foraging events recorded by individual cameras varied depending on species and location in the field.

Together these findings suggest that the choice of the most appropriate method for recording patterns of crop‐foraging depends on the species being studied, with cameras better at predicting researcher data on crop‐foraging by baboons and guards better at predicting researcher data on crop‐foraging by vervets. However, both guards and camera traps appear to be much better at predicting researcher recorded crop loss and frequency of crop‐foraging for baboons than for vervets. Greater temporal changes may make it easier to tease out relative changes in baboon crop‐foraging. Smaller body size (Bolter & Zihlman, 2003; Dechow, 1983) and foraging party sizes (Butynski & de Jong, 2019; Sithaldeen, 2019) of vervets compared to baboons may also mean that guards often miss vervets when they enter the field. Researchers observing this field noticed that guards only responded to vervets in 15% of cases but to baboons in 85% of cases (Findlay & Hill, 2021b). Lower detection of smaller primate species has also been observed in other studies (Wallace, 2010). Biases in camera trap detection may explain their poorer ability to predict vervet crop‐foraging; larger groups are more likely to be detected by cameras, as are larger bodied animals (Kolowski & Forrester, 2017; Pebsworth & LaFleur, 2014; Treves et al., 2010).

We used five cameras in our study, but since each additional camera increases cost and workload, we simulated how reducing the number of cameras on the field would have affected the data collected. The number of crop‐foraging events recorded by subsets of the cameras was influenced by their location. This was more pronounced for vervets, with a 26‐fold difference in the number of crop‐foraging events recorded by the cameras that recorded the most and fewest crop‐foraging events. For both species, the two cameras that recorded most crop‐foraging events were more central on the field edge. The location of the guard is likely to influence this pattern; the two cameras closest to the guard recorded the fewest crop‐foraging events for vervets and the camera closest to the guard was the camera that recorded the second fewest for baboons. Findlay (2016) observed that baboons regularly entered fields at the opposite side to field guards and previous studies have observed similar patterns of guard avoidance by primates (Maples et al., 1976). The researchers, despite observing from a hide, may have also influenced these patterns; for baboons, the camera that recorded the fewest crop‐foraging events was the camera closest to the researcher hide. It is possible that the baboons could sense human presence in the hide, though personal observations suggest that their response to visible observers is much greater than to the occupied hide and they did not show any significant avoidance behaviors such as vigilance, alarm calling, or fleeing. As cameras were close together, they were all a similar distance from salient ecological features such as food and water sources and sleep sites. Researchers and crop guards rather than ecological factors are thus likely to have influenced which cameras collected most images. These findings are likely to be very context‐dependent, varying depending on the surrounding habitat and location of field guards. However, we suggest that placing cameras away from guards is a sensible approach for maximizing the number of crop‐foraging events recorded for any species. Game trails could also be used to help predict where animals are likely to enter fields, a common approach used in camera trapping to increase the likelihood of species detection (Cusack et al., 2015). Using a similar approach, it should be possible for future studies to optimize camera placement to minimize the number of cameras required in the study of anthropogenic foraging.

In future studies using any of the methods presented here, it will be important to consider the pros and cons of each approach. For example, guard literacy and the challenges of liaising with paid guards and their employers may make this a difficult approach in some contexts. Guards may also have other tasks to do on a field making it hard for them to give time to data collection and guards may also miss more crop‐foraging events on larger crop fields.

Camera traps also have limitations; they can only cover a small area relative to human observers, meaning large numbers of cameras may be required to survey large areas. They may also be inappropriate for monitoring flying, burrowing, small or fast moving species (Caravaggi et al., 2020) which may not trigger camera traps as they enter fields. Cost is also a consideration (Glover‐Kapfer et al., 2019); the cameras used in this study cost around 245 GBP each in 2010 when purchased and if more or larger fields need monitoring, then costs may become prohibitive. Animal damage and theft may also further increase the costs of using camera traps (Glover‐Kapfer et al., 2019). Finally, privacy issues may also make cameras undesirable to some farmers or in some locations.

We believe that measures taken in this study (limiting the area surveyed and taking measures to reduce researcher fatigue) mean that the data collected by researchers can be considered reliable and accurate. However, other parameters not considered here, such as group size, may be challenging to accurately assess by researcher observation, particularly if the study species moves rapidly and forages in large groups. Vervets and baboons are generally diurnal (Ayers et al., 2019; Isbell et al., 2017), but researcher observation will be less effective for surveying nocturnal foragers. While these methodological limitations are not exhaustive, they illustrate many of the factors that researchers may need to consider when choosing what method to use to study anthropogenic foraging. The most appropriate method will depend on the local context and study species.

Despite these considerations, we have shown that data from guards and camera traps can predict data collected by researchers and can therefore be a cost‐effective solution for collecting data on crop‐foraging by baboons, and to a lesser extent for crop‐foraging by vervets. The use of both methods in parallel could strengthen any conclusions made about crop‐foraging, as data from one method can cross validate the other. These cost‐efficient methods could allow for the investigation of long‐term trends and the effectiveness of mitigations across multiple sites without the greater investment required by researcher observation. We recommend that researchers carefully consider what questions they wish to address before collecting data by direct observation and assess whether less resource‐intensive methods could be used instead. We also suggest that similar methods to those presented here could be used in other contexts to assess the viability of novel methods for the study of anthropogenic foraging by wildlife.

## CONFLICT OF INTEREST

The authors have no conflict of interest to declare.

## AUTHOR CONTRIBUTIONS

**Ben J. Walton:** Conceptualization (equal); Data curation (lead); Formal analysis (lead); Methodology (equal); Visualization (lead); Writing – original draft (lead); Writing – review & editing (equal). **Leah J. Findlay:** Conceptualization (equal); Investigation (lead); Methodology (equal); Writing – review & editing (equal). **Russell A. Hill:** Conceptualization (equal); Funding acquisition (lead); Supervision (lead); Writing – review & editing (equal).

## Data Availability

Data used in this publication are available from the Dryad Digital Repository https://doi.org/10.5061/dryad.jh9w0vtdf.
